# Modified Oxford technique of colpopexy for the treatment of uterine and vaginal vault prolapse: a retrospective pilot cohort study

**DOI:** 10.3389/fsurg.2023.1222950

**Published:** 2023-06-30

**Authors:** Rajesh Devassy, Antoine Naem, Harald Krentel, Rudy Leon De Wilde

**Affiliations:** ^1^ Dr. Rajesh Devassy’s Centre of Excellence in Gynecological Minimal Access Surgery and Oncology, Dubai London Clinic & Specialty Hospital, Dubai, United Arab Emirates; ^2^ Faculty of Mathematics and Computer Science, University of Bremen, Bremen, Germany; ^3^ Department of Obstetrics, Gynecology, Gynecologic Oncology and Senology, Bethesda Hospital Duisburg, Duisburg, Germany; ^4^ Clinic of Gynecology, Obstetrics and Gynecological Oncology, University Hospital for Gynecology, Pius-Hospital Oldenburg, Medical Campus University of Oldenburg, Oldenburg, Germany

**Keywords:** pelvic organ prolapse, sacrocolpopexy, pectopexy, mersilene, mesh, uterine suspension

## Abstract

**Introduction:**

Pelvic organ prolapse is a common health issue that affects 30.8% of women. Laparoscopic sacrocolpopexy and colpopectopexy are two of the most common procedures to restore the pelvic anatomy. Mesh application on the other hand carries an increased complications risk over the short and long terms. The aim of this study is to provide a basis for meshless sacrocolpopexy and colpopectopexy.

**Methods:**

This study is a retrospective cohort pilot study that analyzed the data of patients with a pelvic organ prolapse according to the pelvic organ prolapse quantification system and underwent the modified technique for sacrocolpopexy and colpopectopexy. Descriptive statistics were used to express the different variables.

**Results:**

A total of 36 patients met the inclusion criteria and provided consent for the participation in this study. The majority of patients were postmenopausal. 22 out of 36 patients received a previous prolapse surgery. All patients presented with reducible vaginal lump. Dyspareunia and sexual dysfunction were the most commonly reported symptoms. The intraoperative complications rate was 0%. Only one patient had a postoperative persistent urinary retention that was managed medically.

**Discussion:**

Sacrocolpopexy and colpopectopexy seems to be a safe alternative to the mesh-based pelvic surgeries with a very low rate of intraoperative complications and favorable follow up outcomes.

## 1. Introduction

Pelvic Organ Prolapse (POP) is the gradual descend of the pelvic structures from its normal anatomical positions to bulge into the vaginal cavity (1–3). This mainly occurs due to a progressive weakening in the pelvic ligaments with a subsequent loss of the apical support (4). The last is thought to predominantly cause anterior and middle compartment prolapse, but also could contribute to a lesser extent to the pathophysiology of the posterior prolapse (5). POP is a common condition affecting about 30.8% of women in the outpatient setting (6). Despite its wide prevalence, it only becomes symptomatic in 2%–3% of patients (7). The incidence of POP surgery peaks in the sixth decade of life, and ranges between 1.5–1.8 per 1,000 women (7, 8). However, with the increase in the living expectancy, it is expected that the number of women with POP and those seeking surgery for POP would increase within the next 20–40 years (9). Patients with symptomatic POP often complain from the sensation of a mass bulging into the vagina, urinary or fecal incontinence, and discomfort during sexual intercourse (10). The consequences of POP extend further to negatively affect the patient's body image and sexuality (11, 12).

Although POP was treated traditionally with hysterectomy (13), there are nowadays more than 40 surgical procedures to restore the normal pelvic anatomy (14). Nevertheless, hysterectomy is not among them anymore because the uterus itself is only a passive structure that has no effect on the prolapse occurrence (15, 16). Sacrocolpopexy was first described in 1957 as attaching the uterus to the sacrum to restore the normal vaginal axis (17). It is now considered the treatment of choice for POP (18, 19), despite the lack of a standardization for this procedure. Currently, laparoscopic sacrocolpopexy is heavily favored over the open approach owing to the comparable outcomes and decreased postoperative morbidity associated with the laparoscopic intervention (19). Another alternative for laparoscopic sacrocolpopexy is laparoscopic colpopectopexy, since it was proven to have comparable outcomes and less postoperative complications compared to sacrocolpopexy (20). One factor that increased the popularity of laparoscopic colpopexy approaches is the ban of transvaginal meshes for POP treatment in April 2019 by the Food and Drug Administration (FDA) due to safety concerns (21). Although the application of surgical meshes seems a safer alternative, a recent study demonstrated that the long-term complications rate of abdominal meshes is around 7%. Those may include pain, dyspareunia, mesh erosion, and small bowel ischemia in some severe cases (22). In light of these events, efforts are being made to eliminate the use of synthetic prostheses (23), or at least to promote the use of lightweight meshes in POP repair surgeries. Therefore, we aim by this paper to describe a modified technique for laparoscopic colpopexy with the use of a double-ended Mersilene suture for the purpose of utero-cervical suspension, and to report the preliminary data of patients who underwent this surgery.

## 2. Material and methods

### 2.1. Study design and setting

This is a single-centre retrospective observational study that included all patients who were admitted for surgical treatment of POP and received the modified Oxford surgical technique for colpopexy at the Dubai London Clinic and Specialty Hospital in Dubai, United Arab Emirates, between November 2017 and December 2021.

### 2.2. Ethical considerations and the patients consent

The study protocol was revised and approved by an independent institutional review board. All patients signed an informed consent for the participation in the study, collecting their medical data and analyzing it for research purposes. This research was conducted in accordance to the ethical standards of the declaration of Helsinki in 1946 and the guidelines of the Committee of Publication Ethics (COPE). This paper was written following the Reporting of studies Conducted using Observational Routinely-collected health Data (RECORD) statement, validated by the Enhancing the Quality and Transparency of Health Research (EQUATOR) network (www.equator-network.org).

### 2.3. Patient allocation and data collection

All included patients had a symptomatic Stage 2 prolapse or higher in any of the three compartments according to the Pelvic Organ Prolapse Quantification (POP-Q) system (24). The prolapse was diagnosed by gynecologic clinical examination and transvaginal ultrasound. Stress urinary incontinence was diagnosed clinically and confirmed through the urodynamic evaluation. Patients who were planning to conceive in the future, refused to receive the modified technique, refused to enroll in this study, had contraindication for general anesthesia, could not tolerate surgery due to chronic illnesses, or who were unwilling to present for at least one follow-up visit were excluded. The choice of sacrocolpopexy or colpopectopexy was done through a joint decision by the patients and the managing gynecologist after thorough clarification and explanation of both procedures. All surgeries were performed by the same surgeon. The patients were followed up postoperatively by re-evaluation during an outpatient visit each 3 months. Data regarding the patients age, symptoms, surgery type, concomitant surgical interventions, intra- and postoperative complications, follow up and recurrences were collected.

### 2.4. Surgical techniques description

#### 2.4.1. Modified sacrocolpopexy technique

**Figure 1:**
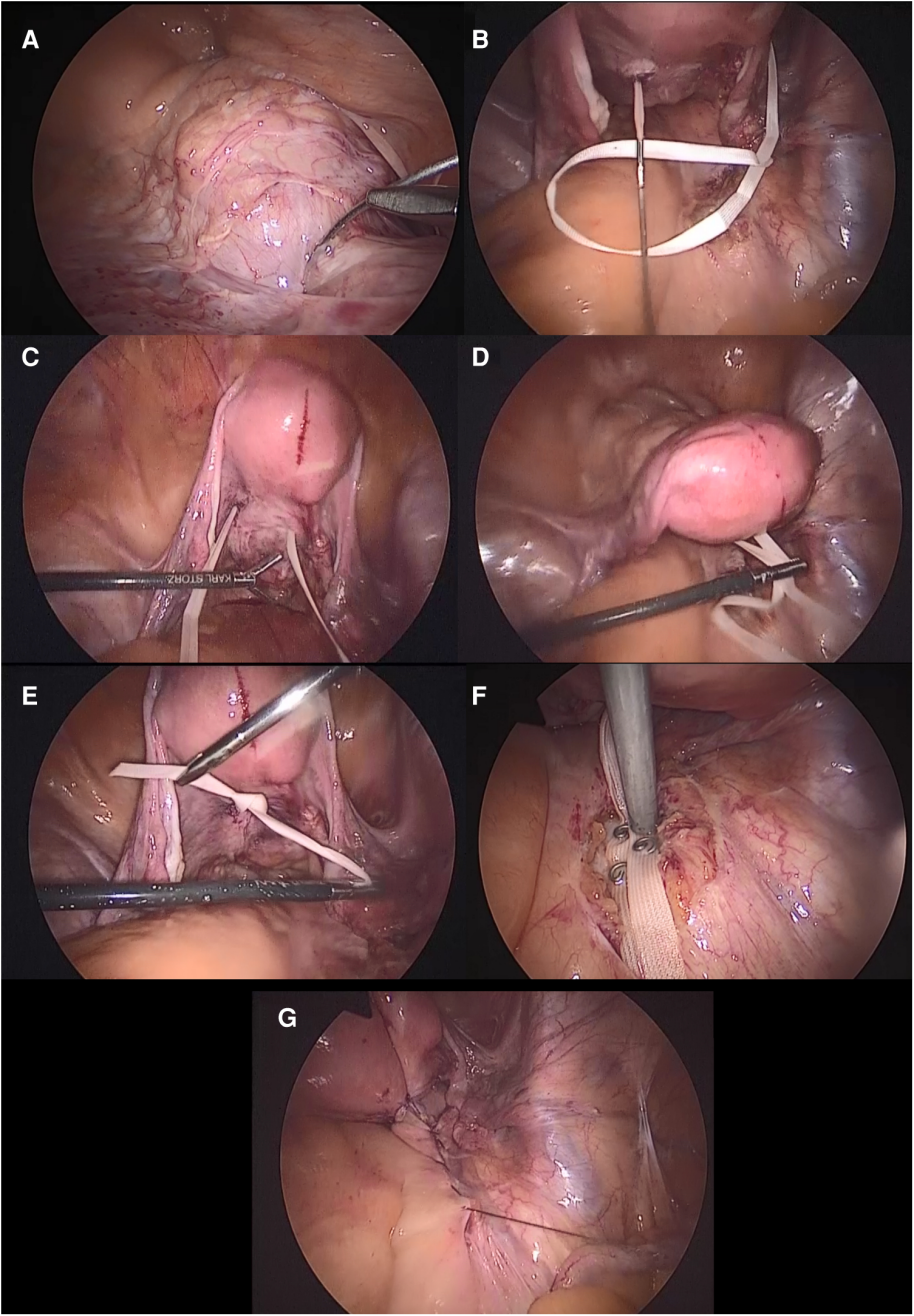
A graphical description of the surgical technique of the modified Mersilene-based sacrocolpopexy. (**A**) The Mersilene suture driven through the left region of the uterine cervix posteriorly. (**B**) The Mersilene suture pulled from the left posterior cervical region. (**C**) The same aforementioned steps repeated on the right side. (**D**) Tension adjustment to ensure a proper prolapse restoration. (**E**) The Mersilene suture knotted over the posterior surface of the uterine cervix. (**F**) The Mersilene suture fixed by permanent tackers on the sacral promontory or permanent polypropylene or polyester sutures. (**G**) Reperitonealization.

After standard patient positioning, abdominal insufflation, and classical laparoscopic ports insertion, the peritoneum over the sacral promontory was incised and the promontory was carefully dissected. The peritoneum was further opened until the retrocervical area was reached. The bladder fold was dissected to ensure an appropriate protection of the urinary bladder and the ureters. Upon completion of this step, the uterine cervix and the upper part of the vagina could be easily recognized. Afterwards, a double-ended Mersilene needle [*RS21—5 mm Mersilene Tape White 1 STRIPX12*″ *(30 cm) BP-1 Double Armed, ©Ethicon US, LLC. 2022. Johnson & Johnson Health Care Systems Inc. 425 NJ-18, Piscataway, NJ 08854, United States*] was introduced into the abdominal cavity. The Mersilene needle was inserted in the anterior left paracervical region (Figure 1A) and driven posteriorly (Figure 1B). The same procedure took place on the right side (Figure 1C). In order to avoid transvaginal suture, an assistant finger was always placed in the vagina to ensure subepithelial isolation. After appropriate tension adjustment to secure a proper suspension (Figure 1D), the suture was knotted and the knot was placed posterior to the uterine cervix (Figure 1E). The suture's ends were then fixed to the sacral promontory with permanent tackers (*ProTack™)* or polypropylene sutures (Figure 1F). The decision between using sutures or permanent tackers was done based on the surgeon's and patient's preference, and the cost covered by the insurance company; since tackers are much more expensive than sutures. Eventually, reperitonealization was done by continuous absorbable suture (Figure 1G).

#### 2.4.2. Modified colpopectopexy technique

**Figure 2:**
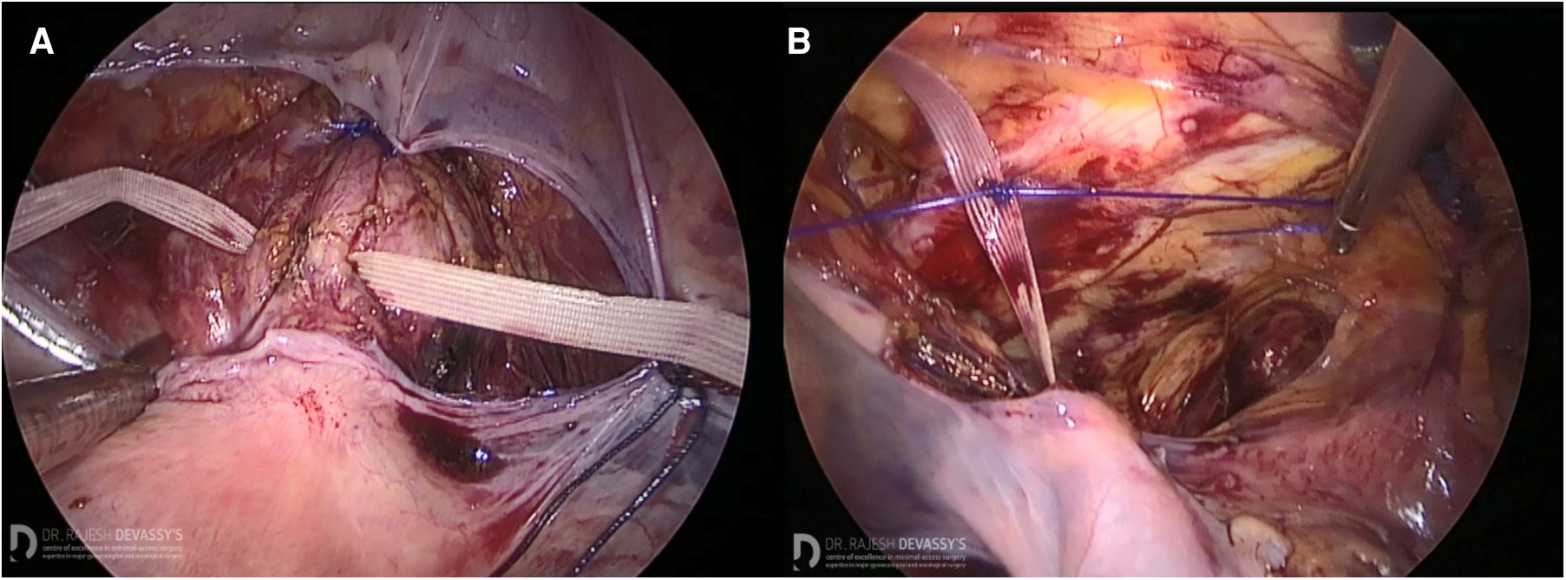
A graphical description of the surgical technique of the modified Mersilene-based colpopectopexy. (**A**) The Mersilene suture driven through the anterior paracervical fascia. (**B**) The Mersilene suture fixed to Cooper's ligament on the left side.

After standard patient positioning, abdominal insufflation, and classical laparoscopic ports insertion, the median umbilical ligament was incised and the dissection was carried out inferiorly until the pubic bone was reached. Thereafter, the dissection was extended laterally to identify Cooper's ligaments bilaterally. The vesico-vaginal space was carefully dissected afterwards to clearly identify the uterine cervix. The double-ended Mersilene needle was introduced into the abdominal cavity and driven transversally through the anterior paracervical fascia (Figure 2A). After intraoperative tension adjustment, the Mersilene suture was fixed to the left Cooper's ligament by a permanent suture (Figure 2B). The same was done on the right side. Eventually, reperitonealization over the suture took place.

### 2.5. Statistical analysis

All data were analyzed by descriptive statistics. Continuous variables were expressed by ranges. Categorical variables were expressed by frequencies and percentages. The statistical analysis was performed using Statistical Package for Social Science (SPSS) software, version 25.0 (SPSS, Chicago, IL).

## 3. Results

A total of 36 patients met the inclusion criteria and provided consent to participate in this study. About one third of the sample (30.5%) fell in the age range 46–55 years. 16 out of 36 patients (44.4%) were postmenopausal. Table 1 demonstrates the patients’ distribution among different age groups. In our series, 10 patients (27.7%) had a previous repair of rectocele, 8 patients (22.2%) had a previous repair of cystocele and 3 patients (8.3%) had prior colposuspension with mesh. There were only 14 patients (38.8%) who did not have a previous POP surgery. 3 patients (8.3%) with prior mesh colposuspension had recurrence. It occurred after 2 years in 1 patient following childbirth, and after 7 and 10 years in the remaining two patients. Upon clinical examination, all patients had a reducible vaginal lump. Urinary incontinence was observed in 34.15% of cases. Sexual dysfunction was reported by two thirds of the patients (60.61%) and dyspareunia was reported by 41.84% of them.

**Table 1:** The patients’ distribution among the different age groups.

Age Group (years old)	*N* (%)
26–35	2 (5.5%)
36–45	7 (19.4%)
46–55	11 (30.5%)
56–65	9 (25%)
66–75	5 (13.8%)
76–85	2 (5.5%)

The modified sacrocolpopexy was performed in 25 patients (69.4%) and the modified colpopectopexy was performed in 11 patients (30.5%). Treatment of cystocele and rectocele using native tissue repair by polypropylene sutures were performed laparoscopically in all cases, besides the modified sacrocolpopexy and colpopectopexy. Table 2 demonstrates the performed procedures. No intraoperative complications were reported, in terms of bowel or bladder injury. Only one patient had a postoperative persistent urinary retention. She was treated with intermittent catheterization for 2 weeks and bladder tonics for a period of 3 months. Thereafter, the bladder shrinkage was reduced and the compartmentalization had disappeared. Erosion or infection were not reported postoperatively by any patient. The mean follow-up time was 3 years, and ranged between 7 months and 5 years.

**Table 2:** The surgical management of the included patients.

Procedure Type	*N* (%)
Modified Sacrocolpopexy	25 (69.4%)
Modified Colpopectopexy	11 (30.5%)
Concomitant Paravaginal native tissue repair	15 (41.6%)
Simultaneous Burch Colposuspension	6 (16.6%)

## 4. Discussion

In this study, we have provided evidence that using a Mersilene suture could be a safe alternative to synthetic meshes in the laparoscopic management of pelvic organ prolapse. The intraoperative complications rate was 0% and only one patient reported urinary retention postoperatively (2.7%). This condition was successfully treated medically. It is noteworthy that major postoperative complications such as erosion, infection, and pelvic pain were not reported by any patient. This is thought to further justify our hypothesis that the presented modified techniques are an alternative to mesh-based sacrocolpopexy and colpopectopexy. Although it is too early to draw conclusions, our sample indicates that these procedures could be helpful for patients with recurrent POP, since the majority of the sample had a previous prolapse surgery. Despite the promising results, it should be noted that this technique could be a feasible but not an easy-to-learn alternative to the traditional approaches. This is mainly attributed to the fact that the dissection of the promontory, pararectal and bladder regions are still needed. Therefore, the same inherent risks of sacrocolpopexy and colpopectopexy should be accounted for. Another drawback of this technique could be the large size of the Mersilene suture, which may hardens its introduction to the abdominal cavity. However, we believe that attaching the Mersilene suture to the cervical and pre-cervical regions is easier and more straightforward than the fixation of traditional meshes to the cervix and upper part of the vagina. On this basis, it is reasonable to postulate that the learning curve of this technique would be higher than the traditional ones. Nevertheless, high suturing skills and precise anatomical knowledge are definitely required for a safe surgery. Of note, a previous study has demonstrated that a surgeon needs to perform 60 laparoscopic sacrocolpopexy to ensure good outcomes and minimal complications rates (25).

The main aim of our modification is promoting native tissue repair and taking a step forward towards a “meshless” pelvic reconstructive surgery. Mesh-based POP surgeries are widely implicated with favorable outcomes, but the short- and long-term mesh-related complications could be the major drawback and a safety concern regarding the use of these prosthetic materials. A recent analysis demonstrated that the complications rate of heavy meshes is 18.8%, which is significantly higher than that of lightweight meshes (2.1%) (22). It is reasonable to consider the use of lightweight meshes instead of the heavier ones, especially when knowing that the recurrence rates were comparable between the two products. However, it should be noted that those complications are serious and worth consideration, especially the ureteric mesh erosion (22). In a different series reporting on the mesh-related complications after the uphold procedure, the erosion and pelvic pain rates were 4% and 5% respectively (26). Another long-term follow up study demonstrated that the 24-month urinary retention rate related to mesh inlay was 2.5% and vaginal adhesions occurred in 1.2% (27). Hospitalization due to mesh-related complications occurred in 7.1% of the followed patients (27). It is already established that some risk factors increase further the risk of vaginal mesh erosion, such as smoking and performing a concomitant total hysterectomy (28). In spite of the protective advantage of supracervical hysterectomy against mesh erosion (29), this procedure could not be always indicated. Therefore, it is quite obvious that the application of abdominal meshes carries inherent risks due to the prosthetic nature of these grafts.

Laparoscopic sacrocolpopexy remains the golden standard for POP surgery (30), as it was reported to carry favorable results in terms of restoring the anterior and middle compartments (31). In their study, Campagna et al. reported that patients undergoing high uterosacral ligament suspension are at 6 times higher risk of prolapse recurrence compared to those who had laparoscopic sacrocolpopexy (8). Similarly, Maher et al. (14) found that vaginal suspension carries an increased risk of subjective failure in comparison with sacrocolpopexy. On the other hand, one randomized controlled trial has demonstrated that both laparoscopic sacrocolpopexy and colpopectopexy carry the same postoperative outcomes in terms of anterior, apical, and posterior objective failure rates (20, 32). It is noteworthy that *de novo* constipation was significantly more reported by patients receiving sacrocolpopexy (20). In another randomized controlled trial comparing sacrocolpopexy to sacrospinous hysteropexy, Van IJsselmuiden et al. (33) found comparable composite success, anatomical and surgical failure rates between the two groups. However, patients who had sacrocolpopexy reported significantly more fecal incontinence compared to the other study group. Bothersome over-active bladder symptoms were also more frequent among patient receiving sacrocolpopexy (33). Those urinary and fecal complications are thought to result from the presacral and pararectal dissection and a subsequent injury to the hypogastric nerves (34, 35). However, it is not clear whether the mesh-associated fibrosis could contribute to those dysfunctions.

Another feasible approach could be the uterosacral ligaments suspension. Some reports showed promising results of this technique when applied laparoscopically or by vaginal natural transluminal endoscopic surgery (36–38).

Moreover, some authors reported the use of the semitendinosus tendon for uterine suspension in sacrocolpopexy and colpopectopexy. This approach sheds light on the use of autologous materials in POP surgery but it is not studied sufficiently yet (39).

Our study has many limitations that should be accounted for. The sample size is small and the follow up period differed among patients. We did not include a control arm to test the differences between the modified and original procedures. In addition, this study was conducted in a tertiary care centre, which makes our sample prone to selection bias. Additionally, some important variables like the timing of surgery, blood loss, and postoperative pain are missing due to the retrospective design of this study. All surgical procedures were performed by the same surgeon. Although it is believed that this would improve the internal validity of this research, it limits its generalizability by making the outcomes and complications rate questionable when this technique is performed by less experienced surgeons, like residents or general gynecologists. On the other hand, the main strength of this research is introducing a new use of the Mersilene suture and highlighting the potential positive outcomes of replacing synthetic meshes with it. Finally, we need to emphasize the fact that this is a pilot study and it is early to draw conclusions about the effectiveness of this technique or recommend standardizing it instead of the current practices. Although our results are promising, future research should consider the differences in operation times, short and long term outcomes and complication rates between the traditional mesh-based surgeries and the Mersilene modified approaches through larger cohort studies and clinical trials.

## 5. Conclusions

The modified technique of sacrocolpopexy and colpopectopexy using a Mersilene suture seems to be a safe alternative to mesh-based pelvic prolapse surgeries. Intraoperative complications were absent and the follow up outcomes were favorable. Future research should investigate the safety, efficacy and the learning curve associated with this modified approach.

## Data Availability

The raw data supporting the conclusions of this article will be made available by the authors, without undue reservation.
